# An Unusual Case: Self-separation of an Idiopathic Epiretinal Membrane

**DOI:** 10.4274/tjo.galenos.2019.62372

**Published:** 2020-03-05

**Authors:** Jale Menteş, Serhad Nalçacı

**Affiliations:** 1 Ege University Faculty of Medicine, Department of Ophthalmology, İzmir, Turkey

**Keywords:** Epiretinal membrane, posterior vitreous detachment, spectral domain optical coherence tomography

## Abstract

Self-separation or peeling of an idiopathic epiretinal membrane (ERM) in an eye with partial posterior vitreous detachment (PVD) is a rare event. A 56-year-old woman presented to our clinic with complaints of floaters in her right eye. Best-corrected visual acuity (BCVA) was 9/10 in this eye. Fundus examination and Spectral domain optical coherence tomography (SD-OCT) revealed an idiopathic ERM and grade 3 PVD in this eye. Four months later, she had complaints of metamorphopsia in her right eye. BCVA was 7/10, while SDOCT images of the right macula were similar to previous images. One week after the last visit, she presented again due to the sudden disappearance of her metamorphopsia complaints. BCVA had improved to 10/10. Fundus examination demonstrated that the ERM had spontaneously separated from the retinal surface as a flap floating in the vitreous and the foveal contour had returned to normal. The etiologic mechanism may be explained as the contracting forces within an immature ERM being stronger than its adhesion to the retina.

## Introduction

Idiopathic epiretinal membrane (ERM) is a vitreoretinal interface disorder of unknown etiology characterized by the formation of a layer of avascular fibrocellular tissue over the internal limiting membrane.^1,2,3,4,5^ Idiopathic ERMs generally occur in individuals over the age of 50 in the absence of any other eye disease and are known to be accompanied by partial or complete posterior vitreous detachment (PVD) in 80-95% of cases.^2,5^

Spontaneous self-separation of an idiopathic ERM from the retinal surface is a rare event. It was reported in the literature that spontaneous separation may occur in eyes with secondary ERM, especially in young patients.^6,7^ However, self-separation of an idiopathic ERM from the retina appearing as a flap in an eye with findings of partial PVD is a very rare phenomenon. 

In this article, we present a case of idiopathic ERM that spontaneously separated from the retinal surface in the form of a flap in an eye with partial PVD, which was immediately followed by resolution of the patients’ visual complaints and anatomic findings.

## Case Report

**Figure 1:**
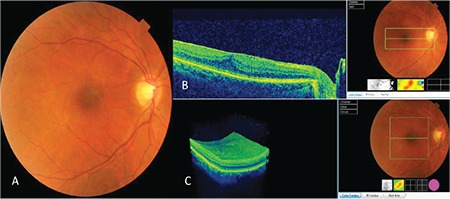
Right eye, idiopathic epiretinal membrane: A) Color fundus photograph, B) B-scan spectral domain optical coherence tomography (SD-OCT) image showing epiretinal membrane and flattening of the foveal contour, C) Threedimensional SD-OCT image

**Figure 2:**
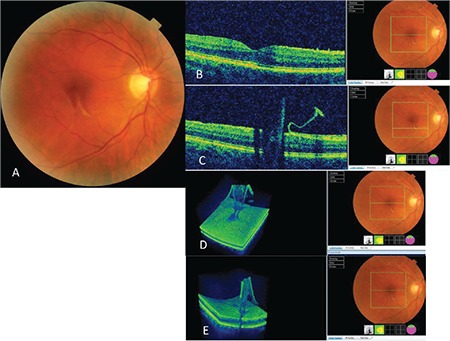
Right eye, idiopathic epiretinal membrane spontaneously detached as a flap: A) Color fundus photograph, B) B-scan spectral domain optical coherence tomography (SD-OCT) image showing recovery of the foveal contour, C) B-scan SD-OCT image showing epiretinal membrane forming a flap with one end freely floating in the vitreous, D, E) Three-dimensional SD-OCT image showing epiretinal membrane floating as a flap in the vitreous

A 56-year-old woman presented with complaints of floaters in her right eye. She had no history of trauma or eye problems and her best corrected visual acuity (BCVA) was 9/10 and 10/10 in the right and left eye, respectively. Intraocular pressure was 16 mmHg in both eyes and anterior segment and fundus examination findings were normal. Spectral domain optical coherence tomography (SD-OCT) (Topcon 3D-OCT, 2000 Corporation, Tokyo, Japan) revealed idiopathic ERM in the macula of the right eye and stage 3 PVD, in which the vitreous is attached only to the optic nerve head. Loss of foveal contour was noted and central macular thickness (CMT) was measured as 360 µm (Figure 1A, B, C). SD-OCT scan of the left eye was normal with no signs of PVD. The patient was followed up with a diagnosis of idiopathic ERM. Four months later, the patient presented with complaints of metamorphopsia. BCVA was 7/10 in her right eye and there was no change in ERM findings on SD-OCT. The vitreous had detached from the optic nerve head, progressing to stage 4 PVD, and CMT was 370 µm. One week later, the patient presented again because her metamorphopsia complaints had suddenly disappeared. BCVA was 10/10 in the right eye. Fundus examination revealed that the ERM had spontaneously detached from the retinal surface and was floating freely in the vitreous in the form of a thin, transparent, grayish-white flap attached to the retina along one side just below the macula. Foveal reflex was completely normal. B-scan and 3D SD-OCT examinations also showed this flap floating in the vitreous with one side still adhering to the retina. Findings pertaining to macular Grade 1 ERM had resolved, the foveal contour had returned to normal, and CMT was 210 µm (Figure 2A, B, C, D). The case was considered spontaneous separation of an idiopathic Grade 1 ERM. At 4-year follow-up, the patient’s BCVA was 10/10. The flap-shaped ERM was slightly contracted and had folded on itself, continuing to float in the vitreous, and CMT was 220 µm. 

## Discussion

Spontaneous ERM separation is an uncommon clinical phenomenon, reported to occur in 1-3% of all ERM cases.^4^ Spontaneous full-thickness, free, or partial flap separations are known to occur in cases of secondary ERM, especially in adolescents and cases of ERM that develop after inflammatory retinal diseases.^5,6,7^ However, the self-separation of an idiopathic ERM from the retinal surface is rare.^2,3,4,5^ In this article, we present an eye with partial PVD which developed an idiopathic ERM that later spontaneously separated from the retinal surface as a flap. In their series of 1248 idiopathic ERM cases, Yang et al.^3^ determined with SD-OCT that the prevalence of spontaneous separation in eyes with and without PVD was 1.5% and 13.4%, respectively, during the 33-month follow-up period. Meyer et al.^6^ reported spontaneous separation rates of 0.47 and 2.3% in eyes with and without PVD, respectively, during 2-13 months of follow-up in their series of 210 patients under the age of 30 who had idiopathic ERM. Our patient had stage 3 PVD at initial examination.

An interesting and previously unreported finding in the current case is that the base of the self-separated ERM flap was positioned inferiorly and toward the peripheral retina, as with peripheral retinal tears. In earlier cases of ERM with partial spontaneous separation described in the literature, whether idiopathic or secondary, the base of the flap is usually positioned toward the optic disc or the temporal or superior directions.^3,6^ In a study reporting decreased CMT and increased BCVA following spontaneous idiopathic ERM separation, it was determined that new defects may form at the retinal inner segment/outer segment (IS/OS) line after spontaneous separation and that ERM recurrence is more common in such cases, emphasizing that the IS/OS line is more susceptible to vertical forces.^6^ The absence of any defects in the retinal layers in our patient could be attributed to the retinal traction being weak and of short duration.

Two mechanisms have been proposed in the literature to explain the pathogenesis of spontaneous ERM separation. The first and most common mechanism is a process that occurs due to the induction of PVD.The ERM may spontaneously detach when the contractive forces within an immature ERM are stronger than its adhesion to the retina. The second mechanism is a process resulting from the tangential traction created by the cells in the inner retinal layers that occur in eyes in which PVD has already developed.^5,6^

We believe that the spontaneous idiopathic ERM detachment in our patient is consistent with the first mechanism. In our opinion, the likely mechanism is that the anteroposterior tractional forces in the concentrated vitreous overcome the tangential tractional forces, and due to this force, the base of the already thin and immature ERM detaches from the retina in the form of a flap in the periphery.
